# Efficacy and Safety of TACE Combined With Lenvatinib Plus PD-1 Inhibitors Compared With TACE Alone for Unresectable Hepatocellular Carcinoma Patients: A Prospective Cohort Study

**DOI:** 10.3389/fonc.2022.874473

**Published:** 2022-04-21

**Authors:** Shuping Qu, Xiaobing Zhang, Yutian Wu, Yan Meng, Hongyu Pan, Qiang Fang, Lei Hu, Jin Zhang, Ruoyu Wang, Lixin Wei, Dong Wu

**Affiliations:** ^1^ Department of Hepatic Surgery, Third Affiliated Hospital of Second Military Medical University, Shanghai, China; ^2^ Department of Hepatic Surgery, The First Hospital of Putian, Putian, China; ^3^ Tumor Immunology and Gene Therapy Center, Third Affiliated Hospital of Second Military Medical University, Shanghai, China

**Keywords:** hepatocellular carcinoma, transcatheter arterial chemoembolization, lenvatinib, programmed cell death-1 inhibitor, neutrophil-to-lymphocyte ratio

## Abstract

**Purpose:**

To compare the efficacy and safety of the combination of transcatheter arterial chemoembolization (TACE), Lenvatinib, and programmed cell death protein-1 (PD-1) inhibitors (combination group) with TACE (TACE group) in the treatment of patients with unresectable hepatocellular carcinoma (uHCC).

**Methods:**

We consecutively enrolled 110 patients with uHCC in this prospective cohort study, with 56 patients receiving combination treatment and 54 patients receiving TACE from November 2017 to September 2020. The differences in tumor response, survival benefit, and adverse events (AEs) were compared between the two groups. Factors affecting survival were identified *via* Cox regression analysis.

**Results:**

Compared with the TACE group, the combination group had a higher objective response rate (ORR) (67.9% vs. 29.6%, p < 0.001), longer median progression-free survival (mPFS) (11.9 vs. 6.9 months, P = 0.003) and overall survival (mOS) (23.9 vs. 15.3 months, p < 0.001). Multivariate analysis showed that the neutrophil-to-lymphocyte ratio (NLR) and the treatment option were independent factors associated with the PFS and OS. Further subgroup analysis showed that patients with low NLR (≤median 3.11) receiving combination therapy had better mPFS (20.1 vs. 6.2 months, P < 0.001) and mOS (28.9 vs. 15.2 months, P < 0.001) than those receiving TACE, while no obvious difference in PFS or OS was observed between the two groups in patients with high NLR (> 3.11). There were no unexpected toxicities in the combination group.

**Conclusion:**

Compared with TACE, the combination treatment demonstrated an improved clinical efficacy and manageable safety profile in patients with uHCC. Combination treatment showed better therapeutic efficacy in patients with low NLR; therefore, this ratio could be used to identify patients who will benefit from this treatment.

## Introduction

Hepatocellular carcinoma (HCC) is the fifth most common cancer and the second leading cause of cancer-related death worldwide (1). Approximately 70%–80% of patients with HCC are diagnosed at the intermediate-advanced stage (2), and only candidates for palliative treatments such as chemotherapy, transcatheter arterial chemoembolization (TACE), radiotherapy or sorafenib, resulting in a dismal prognosis. Recently, little improvements in overall survival (OS) with monotherapy have been noted despite the rapid evolution of systemic agents in patients with uHCC (3, 4). Therefore, there is currently an unmet need to explore more effective treatment modalities with improved antitumoral efficacy in patients with uHCC.

TACE, which is the first therapeutic modality to provide survival benefits in patients with uHCC (5), is the standard treatment for BCLC stage B HCC in Western countries (6) and stage B and C in China (7). In the real-world BRIDGE study that included 18,031 patients from 14 countries, TACE was identified as the first-line treatment for approximately 50% of patients at the BCLC C stage of HCC (2). Also, some previous studies have demonstrated that TACE therapy alone could be of more benefit to patients at the BCLC C stage of HCC than the best supportive care (8, 9). Some studies even demonstrated that TACE could achieve a comparable survival outcome to sorafenib in BCLC stage C HCC patients (10, 11). TACE was also found to promote the immunogenic cell death of cancer cells (12) and induce the release of the tumor-associated antigen (13), enabling more antitumor activity of immune checkpoint inhibitors (ICIs). Furthermore, The upregulated expression of Vascular Endothelial Growth Factor (VEGF) and Fibroblast Growth Factor (FGF) (14) by TACE could be effectively inhibited by tyrosine kinase inhibitors(TKIs) in a preclinical study (15), leading to better clinical outcomes combined with TKIs (16, 17). Thus, TACE may hold a greater promise in the context of rapid advances in systemic therapy.

Several recent studies have demonstrated that the combination of ICIs and antiangiogenic agents exhibits superior antitumor activity and a significantly improved survival compared to monotherapies. In the IMbrave150 trial (18), the combination of atezolizumab and bevacizumab showed better tumor response and survival than sorafenib, leading to accelerated Food and Drug Administration approval for advanced HCC. Similarly promising efficacy was also reported in the KEYNOTE 524 trial (19) that was based on pembrolizumab plus Lenvatinib and demonstrated a mOS of 22 months, a mPFS of 9.3 months, and an objective response rate (ORR) of 46% per modified Response Evaluation Criteria in Solid Tumors (mRECIST). However, it should be noted that clinical benefits from such combinations with systemic therapies remain limited to a subset of patients because of the high proportion of primary drug resistance.

Considering the potential synergistic effect of the combination of TACE, Lenvatinib, and PD-1 inhibitors, we conducted this prospective cohort study to compare the safety and efficacy of the triple combination treatment with those of TACE alone for patients with uHCC.

## Patients and Methods

### Patients

This prospective cohort study was conducted at the Department of Hepatic Surgery, Third Affiliated Hospital of the Second Military Medical University, Shanghai, China, from November 2017 to September 2020.The inclusion criteria were as follows: patients were diagnosed with HCC by histopathological biopsy or clinical features according to the American Association for the Study of Liver Diseases guidelines; lesions not amenable to curative resection evaluated by surgeons due to an insufficient future liver remnant, extensive or multifocal bi-lobar tumors, extrahepatic metastasis, and major vascular invasion (20); Eastern Cooperative Oncology Group (ECOG) performance status (PS) 0 or 1; Child-Pugh class A or B7; accept heart, kidney and bone marrow function; at least one measurable target lesion by mRECIST; no prior systemic therapy for liver cancer. Patients were excluded if they had any of the following conditions: life expectancy was less than 3 months; patients simultaneously received other forms of therapy such as radiotherapy and ablation; uncontrollable ascites; decompensated liver cirrhosis; concomitant with other primary malignancies. The study protocol was approved by the ethical committee of the Third Affiliated Hospital of the Second Military Medical University. Written informed consent was obtained from all patients.

### TACE Procedure

The vascular catheter was inserted through a femoral artery using the Seldinger technique to the hepatic artery, then hepatic angiography was performed. The catheter tip was inserted selectively into the tumor-feeding artery and the pirarubicin manually emulsified with iodized oil was injected into these vessels, followed by embolization with absorbable gelatin sponge particles. TACE was conducted repeatedly on demand according to investigators’ consideration, mainly based on the proportion of viable tumors and status of hepatic function.

### Medication Treatment

All patients were discussed and evaluated by multidisciplinary teams. The TACE-Lenvatinib-PD-1 inhibitor treatment strategy was recommended by physicians if the patient was suitable for the combination therapy. Also, the patient was fully informed about the efficacy and cost of the drugs and the potential adverse effects (AEs). If the patient agreed to the physician’s recommendation, Lenvatinib and PD-1 inhibitors were administered, otherwise, the patient received TACE treatment alone.

Lenvatinib was administered one week prior to the first TACE procedure. Patients received Lenvatinib at a dose of 12 mg/d if they weighed ≥60 kg and 8 mg/d if they weighed <60 kg. Lenvatinib was interrupted for two days before and after each TACE session if no obvious symptoms occurred after TACE. Dose modifications and discontinuation were permitted according to the label.

Patients received 200 mg pembrolizumab or 240 mg toripalimab intravenously every three weeks based on their labels. PD-1 inhibitors were administered one day prior to the first TACE. Discontinuation was allowed if patients experienced severe AEs. Each cycle of combination therapy was defined as four doses of PD-1 inhibitors and 1–2 episodes of TACE.

Patients discontinued the combination treatment in the case of unacceptable toxicity or disease progression.

All patients with hepatitis B virus (HBV) infection received antiviral treatment initially and continued antiviral treatment throughout the treatment period.

### Efficacy Assessment

The primary endpoints were the OS. The OS was defined as the time from treatment initiation to death of any cause. The secondary endpoints were the PFS, the ORR and safety profiles. The PFS was defined as the time from treatment initiation to disease progression according to mRECIST or death of any cause. The mRECIST assessment is a method of evaluating the therapeutic response based on the viable tumor by an enhanced scan in HCC (21). The objective response rate (ORR) was calculated as the sum of the complete response (CR) and partial response (PR). The disease control rate (DCR) was the sum of the CR, PR, and stable disease (SD). Early tumor shrinkage (ETS) was defined as tumor shrinkage at the first radiologic evaluation (approximately eight weeks after treatment initiation) in the sum of target lesions’ longest diameters from baseline according to the Response Evaluation Criteria in Solid Tumors (RECIST) (22).

### Safety Assessment

Safety was evaluated *via* vital signs, clinical laboratory testing, and an assessment of the incidence and severity of AEs according to the National Cancer Institute Common Terminology Criteria for Adverse Events (NCI-CTCAE) version 5.0.

### Follow-up

All patients were monitored regularly. Followed-up assessments were conducted every 6–8 weeks. Each session consisted of tumor response assessment by enhanced computed tomography (CT) or magnetic resonance imaging (MRI) and laboratory tests including blood and urine routine, liver and kidney function tests, thyroid function tests, HBV-DNA, and tumor markers, including alpha-fetoprotein (AFP) and des-gamma-carboxy prothrombin (DCP) in the two groups. An additional myocardial enzymology examination was necessary in the combination group. In addition, chest X-ray was also regular test. ^18^F-fluorodeoxyglucose positron emission tomography/computed tomography (FDG-PETCT) examination was performed if necessary.

### Statistical Analysis

Statistical analyses were performed using STATA version 15 for Windows. Categorical variables were compared using the χ^2^ test or Fisher’s exact test. Continuous variables were compared using either the t-test for normally distributed variables or the Mann–Whitney U test for variables with a non-normal distribution. The median NLR value was chosen as the cutoff to divide the data into high and low subgroups. The survival analysis was carried out using the Kaplan-Meier method and compared using the log-rank test. All variables with p < 0.1 during univariate analyses were included in multivariate analyses, which included a Cox regression analysis to identify factors independently associated with PFS and OS. P < 0.05 was considered statistically significant.

## Results

### Baseline Characteristics

**Figure 1:**
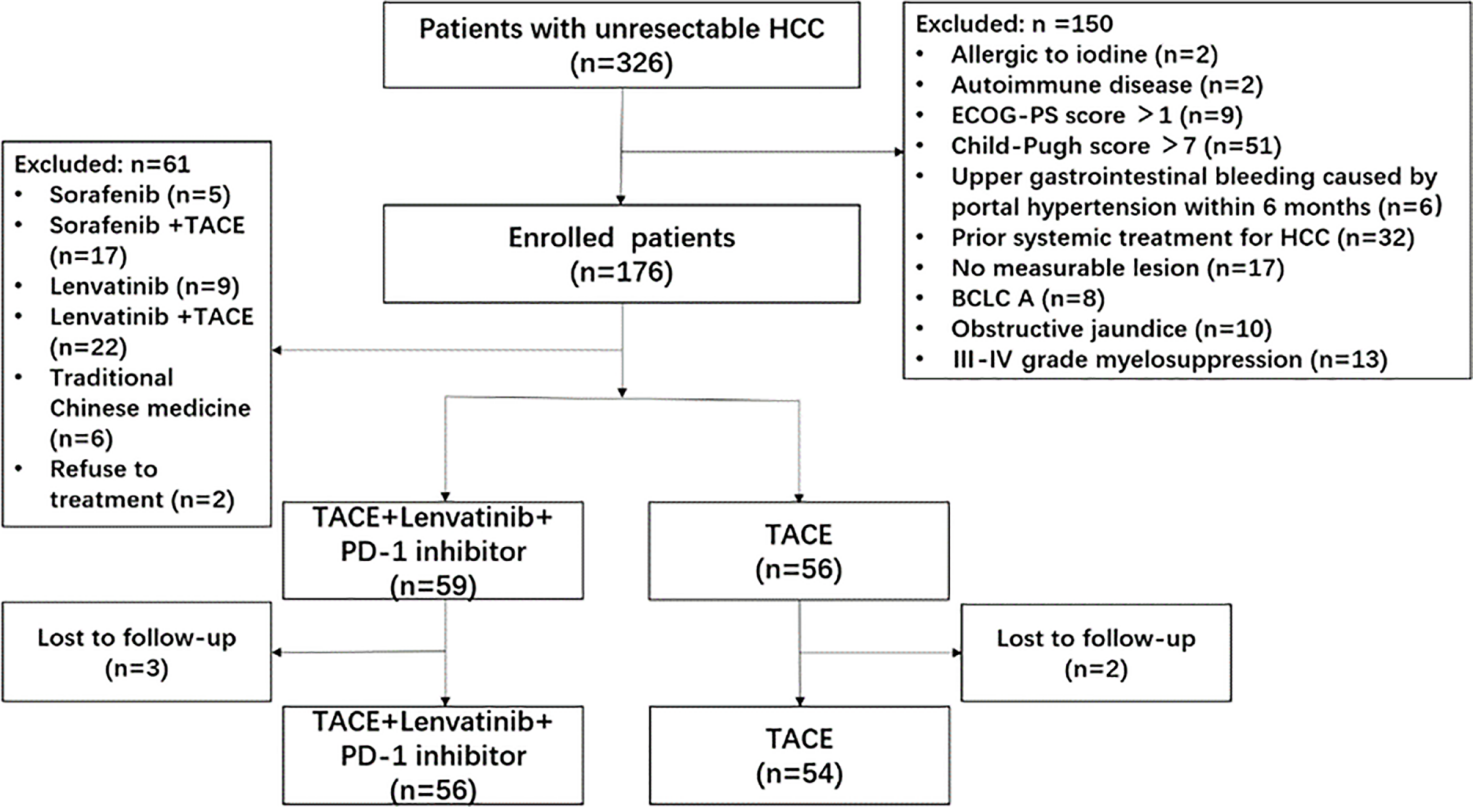
Flowchart of the patient selection process. HCC, hepatocellular carcinoma; ECOG-PS, Eastern Cooperative Oncology Group performance status; BCLC, Barcelona Clinic Liver Cancer; TACE, transcatheter arterial chemoembolization; PD-1, programmed cell death-1.

From November 2017 to September 2020, 326 patients with uHCC were recruited and 216 patients were excluded. Finally, 110 patients were enrolled in this study and were observed either until death or the last follow-up date (Mar 31, 2021) for living patients. Fifty-six patients received combination therapy and 54 patients received TACE alone (**Figure 1**). Nine patients received pembrolizumab while the other 47 patients received toripalimab in the combination group. There was no statistically significant difference between the baseline characteristics of the patients in both groups, except for the age that was slightly younger in the combination group (**Table 1**). At the time of data cutoff, the median duration of follow-up was 21.3 months (range, 9.3-38.5) in the combination group and 12.4 months (range, 3.5-19.5) in the TACE group. The median Lenvatinib treatment duration was 10.7 months (range, 2.4-35.0) and the median number of PD-1 inhibitor administrations was 11.0(range, 2-32). One hundred and thirty-six TACE courses were performed in the combination group (a mean of 2.4 procedures, range: 1–4) and 160 TACE courses were performed in the TACE group (a mean of 3.0 courses, range, 2–4). Patients with TACE alone received more TACE courses compared with those in the combination group (P = 0.001).

**Table 1:** Baseline demographic and clinical characteristic of enrolled patients.

Characteristics	Combination Group (N = 56)	TACE Group (N = 54)	P value
Gender			1.000
Female	5 (8.9%)	5 (9.3%)	
Male	51 (91.1%)	49 (90.7%)	
Age(years), median (range)	51 (24 – 82)	55 (29 – 80)	0.041
≤55	37 (66.1%)	28 (51.9%)	0.129
>55	19 (33.9%)	26 (48.1%)	
Hypertension			0.519
Yes	6 (10.7%)	8 (14.8%)	
No	50 (89.3%)	46 (85.2%)	
Etiology			0.718
Hepatitis B	43 (76.8%)	43 (79.6%)	
Non-B Non-C	13 (23.2%)	11 (20.4%)	
ECOG PS			0.535
0	49 (87.5%)	45 (83.3%)	
1	7 (12.5%)	9 (16.7%)	
Child-Pugh score			0.995
5	45 (80.3%)	43 (79.6%)	
6	8 (14.3%)	8 (14.8%)	
7	3 (5.4%)	3 (5.6%)	
ALBI grade			0.088
1	34 (60.7%)	24 (44.4%)	
2	22 (39.3%)	30 (55.6%)	
BCLC stage			0.738
B	17 (30.4%)	18 (33.3%)	
C	39 (69.6%)	36 (66.7%)	
AFP(ng/mL), median(Q1, Q3)	94.6 (4.1 – 1956.8)	336.5 (25.2 – 2029.3)	0.176
≤ 400	34 (60.7%)	30 (55.6%)	0.583
>400	22 (39.3%)	24 (44.4%)	
DCP(mAU/mL), median(Q1, Q3)	377.5 (40.0 – 7984.0)	818.0 (306.8 – 1312.3)	0.246
≤ 664	32(57.1%)	23 (42.6%)	0.127
> 664	24 (42.9%)	31 (57.4%)	
Target tumor size (cm)			0.703
≤ 7.5	27 (48.2%)	28 (51.9%)	
> 7.5	29 (51.8%)	26 (48.1%)	
Target tumor numbers			0.367
1	13 (23.2%)	12 (22.2%)	
2	37 (66.1%)	31 (57.4%)	
>=3	6 (10.7%)	11 (20.4%)	
MVI			0.890
Absence	37 (66.1%)	35 (64.8%)	
Presence	19 (33.9%)	19 (35.2%)	
EHS			0.565
Absence	27 (48.2%)	29 (53.7%)	
Presence	29 (51.8%)	25 (46.3%)	
BMI (kg/m^2^), median (Q1, Q3)	23.11 (20.78 – 25.06)	22.90 (20.98 – 25.27)	0.484
PLT(x10^9^/L), median (Q1, Q3)	182 (142 – 221)	156 (118 – 215)	0.278
NLR, median (Q1, Q3)	3.22 (2.30 – 4.23)	2.98 (2.01 – 3.86)	0.374
ALT(u/L), median (Q1, Q3)	29 (18 – 46)	33 (23 – 57)	0.219
ALP(u/L), median (Q1, Q3)	125 (78 – 162)	108 (86 – 161)	0.645
PT (sec), median (Q1, Q3)	11.8 (11.3 – 12.6)	12.0 (11.2 – 12.6)	0.582
TB(μmol/L), median (Q1, Q3)	13.5 (10.7 – 18.6)	14.5 (12.0 – 21.1)	0.304
ALB(g/L), median (Q1, Q3)	40.6 (36.9 – 42.8)	40.0 (36.6 – 42.0)	0.312
Cr(μmol/L), median (Q1, Q3)	75.0 (66.3 – 85.8)	72.5 (66.0 – 80.0)	0.354

Data are presented as n (%) or median (Q1, Q3), Q1 and Q3 are 25th percent and 75th percent of interquartile range.

ECOG-PS, Eastern Cooperative Oncology Group performance status; ALBI grade albumin-bilirubin grade; BCLC, Barcelona Clinic Liver Cancer; AFP, alpha-fetoprotein; DCP, Des-gamma carboxy prothrombin; MVI, macroscopic vascular invasion; EHS, extrahepatic spread; BMI, body mass index; PLT, platelet; NLR, neutrophil-to-lymphocyte ratio; ALP, alkaline phosphatase; PT, prothrombin time; TB, total bilirubin; ALB, albumin; Cr, creatinine.

### Tumor Response

The best tumor response rates are shown in **Table 2**. The response rates of patients, according to mRECIST, differed significantly between the combination group and the TACE group. The ORR in the combination group was significantly higher than that in the TACE groups (67.9% vs. 29.6%, p < 0.001). The DCR in the combination group was numerically higher than that in the TACE group; however, the difference was not statistically significant (92.9% vs. 83.3%, p = 0.122). Among the 54 patients treated with TACE alone, ETS ≥ 10% was achieved in 11 (20.4%) patients while in the combination group, the higher rate of ETS ≥ 10% was achieved in 29 (51.8%) patients.

**Table 2:** Best tumor response according to the mRECIST.

Characteristics	Combination Group (N = 56) No. (%)	TACE Group (N = 54) No. (%)	P value
CR	11 (19.6%)	2 (3.7%)	0.010
PR	27 (48.2%)	14 (25.9%)	
SD	14 (25.0%)	29 (53.7%)	
PD	4 (7.1%)	9 (16.7%)	
ORR	38 (67.9%)	16 (29.6%)	<0.001
DCR	52 (92.9%)	45 (83.3%)	0.122

Data are presented as n (%).

CR, complete response; PR, partial response; SD, stable disease; PD, progressive disease; ORR, objective response rate; DCR, disease control rate; mRECIST, modified Response Evaluation Criteria In Solid Tumors.

### Survival Assessment

**Figure 2:**
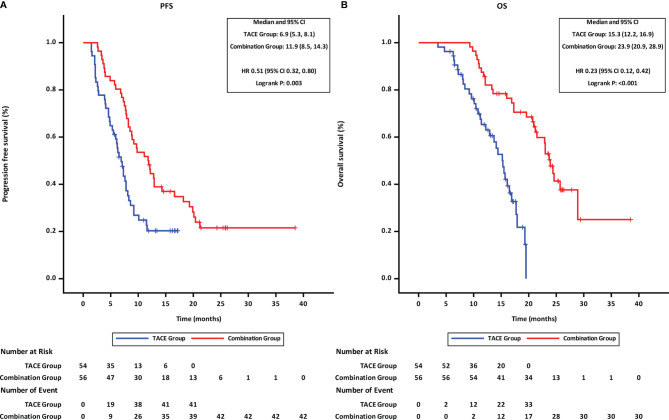
Progression-free and overall survival of patients receiving the different treatments. **(A)** progression-free survival. **(B)** overall survival. TACE, transcatheter arterial chemoembolization; PD-1, programmed cell death-1; HR, hazard ratio; CI, confidence interval.

At the time of last follow-up date (Mar 31,2021), 42 patients were found disease progression and 30 patients had died in the combination group, while the disease was found to have progressed in 41 patients and 33 patients had died in the TACE group. Both PFS and OS of patients in the combination group were better than those of patients in the TACE group. The mPFS was 11.9 months for patients in the combination group as compared to 6.9 months for patients in the TACE group (p = 0.003, HR = 0.51, 95% CI 0.32–0.80; **Figure 2A**). Similarly, the mOS was significantly higher for patients in the combination group than that in the TACE group (23.9 vs. 15.3 months; p < 0.001, HR = 0.23, 95% CI 0.12–0.42; **Figure 2B**).

### Prognostic Factors for PFS and OS

The factors influencing PFS by univariate and multivariate analysis are shown in **Table 3**. Univariate analysis showed that PFS was associated with NLR (≤ vs. > 3.11) and the treatment option (Combination therapy vs. TACE). On the multivariate analysis, NLR (≤ vs. > 3.11) and treatment option (Combination therapy vs. TACE) were also identified as independent prognostic factors of PFS.

**Table 3:** | Univariate and multivariate analyses factors associated with progression-free survival.

Variables	Univariate analysis		Multivariate analysis	
	HR (95%CI)	P1	HR (95%CI)	P2
Gender	0.55	0.163		
female vs. male	(0.24, 1.27)			
Age, year	1.02	0.932		
> vs. ≤ 55	(0.66, 1.58)			
ECOG-PS	0.78	0.415		
0 vs. 1	(0.42,1.43)			
NLR	**0.52**	**0.004**	**0.54**	**0.006**
≤ vs. > 3.11	**(0.33, 0.81)**		**(0.34, 0.84)**	
ALBI grade	0.80	0.319		
1 vs. 2	(0.52, 1.24)			
Child-Pugh	1.24	0.456		
5 vs. 6 & 7	(0.71, 2.17)			
AFP, ng/ml	0.78	0.264		
≤ vs. > 400	(0.50, 1.21)			
DCP, mAU/ml	0.71	0.119		
≤ vs. > 664	(0.46, 1.09)			
Target tumor size, cm	1.23	0.346		
≤ vs. > 7.5	(0.80, 1.90)			
Target tumor number	0.85	0.553		
1 vs. ≥ 1	(0.51, 1.44)			
MVI	0.70	0.112		
absent vs. present	(0.45, 1.09)			
EHS	0.94	0.793		
absent vs. present	(0.61, 1.46)			
BCLC stage	0.94	0.798		
B vs. C	(0.59, 1.50)			
Treatment option	**0.51**	**0.003**	**0.52**	**0.005**
Combination therapy vs. TACE	**(0.32, 0.80)**		**(0.33, 0.82)**	

The bold values highlighted the factors with significant difference.

P1 value was calculated with log-rank test. Any variables that were statistically significant at P < 0.1 in the univariate analysis were used in multivariate analyses using Cox regression analysis.

P2 value was calculated by multivariable Cox proportional-hazards analysis.

ECOG-PS, Eastern Cooperative Oncology Group performance status; NLR, neutrophil-to-lymphocyte ratio; ALBI grade albumin-bilirubin grade; AFP alpha-fetoprotein; DCP, Des-gamma carboxy prothrombin; MVI, macrovascular invasion; EHS, extrahepatic spread; BCLC, Barcelona Clinic Liver Cancer.

The factors influencing the OS identified *via* univariate and multivariate analyses of the factors are shown in **Table 4**. Univariate analyses revealed that the OS was significantly associated with the NLR (≤ vs. > 3.11), AFP (≤ vs.> 400 ng/ml), DCP (≤ vs.> 664 mAU/ml), macroscopic vascular invasion (MVI) (absent vs. present) and treatment option (combination therapy vs. TACE). Upon multivariate analysis, NLR (≤ vs.> 3.11), AFP (≤ vs.> 400 ng/ml), MVI (absent vs. present), and treatment option (combination therapy vs. TACE) were identified as independent prognostic factors of OS.

**Table 4:** Univariate and multivariate analyses of factors associated with overall survival.

Variables	Univariate analysis		Multivariate analysis	
	HR (95%CI)	P1	HR (95%CI)	P2
Gender female vs. male	0.62 (0.22, 1.71)	0.355		
Age, year > vs.≤ 55	1.26 (0.77, 2.07)	0.358		
ECOG-PS 0 vs. 1	0.77 (0.40, 1.48)	0.428		
NLR ≤ vs. > 3.11	**0.51 (0.30, 0.86)**	**0.012**	**0.52 (0.31, 0.89)**	**0.016**
ALBI grade 1 vs. 2	0.86 (0.52, 1.41)	0.551		
Child-Pugh 5 vs. 6 & 7	0.92 (0.50, 1.69)	0.782		
AFP, ng/ml ≤ vs. > 400	**0.51 (0.31, 0.85)**	**0.009**	**0.49 (0.29, 0.83)**	**0.008**
DCP, mAU/ml ≤ vs. > 664	**0.61 (0.37, 1.00)**	**0.048**	0.90 (0.53, 1.54)	0.699
Target tumor size, cm ≤ vs. > 7.5	0.94 (0.57, 1.54)	0.807		
Target tumor number 1 vs. ≥ 1	1.02 (0.56, 1.85)	0.949		
MVI absent vs. present	**0.63 (0.38, 1.05)**	**0.076**	**0.57 (0.34, 0.98)**	**0.041**
EHS absent vs. present	0.82 (0.50, 1.36)	0.449		
BCLC stage B vs. C	0.96 (0.56, 1.65)	0.8912		
Treatment option Combination therapy vs. TACE	**0.23 (0.12, 0.42)**	**<0.001**	**0.18 (0.10, 0.35)**	**<0.001**

The bold values highlighted the factors with significant difference.

P1 value was calculated with log-rank test. Any variables that were statistically significant at P < 0.1 in the univariate analysis were used in multivariate analyses using Cox regression analysis.

P2 value was calculated by multivariable Cox proportional-hazards analysis.

ECOG-PS, Eastern Cooperative Oncology Group performance status; NLR, neutrophil-to-lymphocyte ratio; ALBI grade albumin-bilirubin grade; AFP alpha-fetoprotein; DCP, Des-gamma carboxy prothrombin; MVI, macrovascular invasion; EHS, extrahepatic spread; BCLC, Barcelona Clinic Liver Cancer.

### Subgroup Analysis of Factors Associated With Patients’ Survival

**Figure 3:**
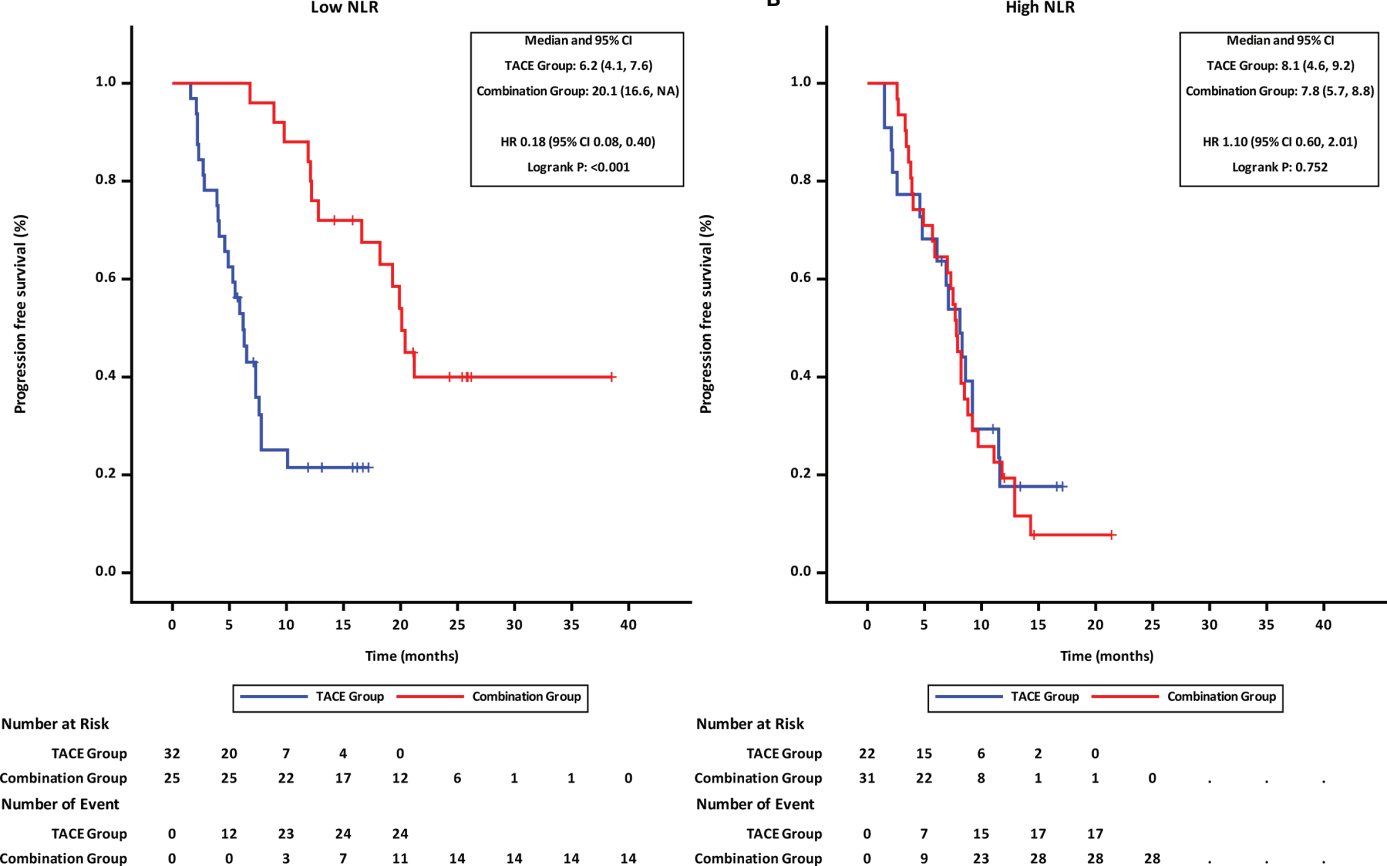
Progression-free survival of groups of patients with different NLR statuses. **(A)** low NLR. **(B)** high NLR. TACE, transcatheter arterial chemoembolization; PD-1 programmed cell death-1; NLR, neutrophil-to-lymphocyte ratio; HR, hazard ratio; CI, confidence interval.

**Figure 4:**
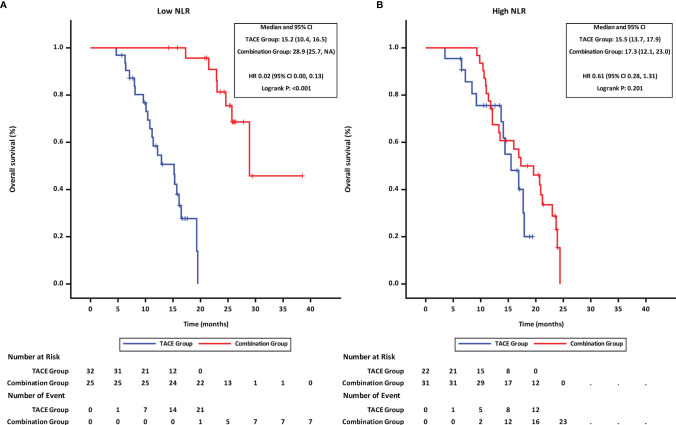
Overall survival of groups of patients with different NLR statuses. **(A)** low NLR. **(B)** high NLR. TACE, transcatheter arterial chemoembolization; PD-1, programmed cell death-1; NLR, neutrophil-to-lymphocyte ratio; HR, hazard ratio; CI, confidence interval.

Patients were stratified into two groups by the NLR value (≤3.11 and >3.11; with 3.11 being the median). Subgroup analyses revealed that in patients with low NLR (≤3.11), the PFS (**Figure 3A**) and OS (**Figure 4A**) in the combination group were significantly prolonged compared with those in the TACE group, while no statistically significant differences in PFS (**Figure 3B** **)** and OS (**Figure 4B**) were found in patients with high NLR (>3.11) between the two groups.

**Figure 5:**
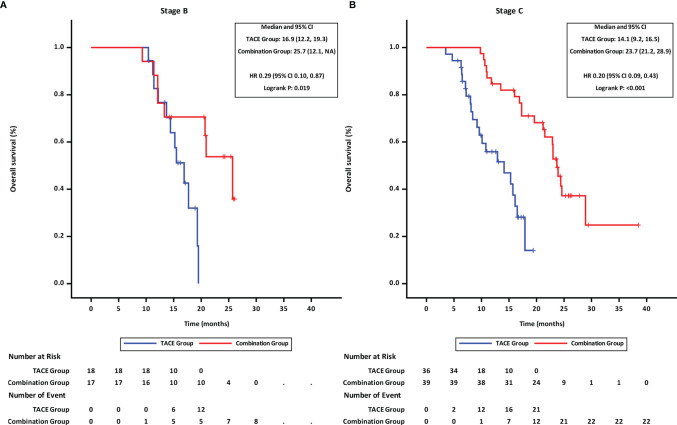
Overall survival of groups of patients at different BCLC stages. **(A)** BCLC stage B, **(B)** BCLC stage C TACE, transcatheter arterial chemoembolization; PD-1, programmed cell death-1; BCLC, Barcelona Clinic Liver Cancer; HR, hazard ratio; CI, confidence interval.

Further, the effects of treatment options and tumor-related factors on survival were analyzed. The OS in the combination group was longer than that in TACE group irrespective of the tumor stage (mOS of BCLC stage B: 25.7 months in the combination group vs. 16.9 months in the TACE group, HR = 0.29,95%CI 0.10–0.87, p = 0.019 (**Figure 5A**); mOS of BCLC stage C: 23.7 months in the combination group vs. 14.1 months in the TACE group, HR = 0.20,95%CI 0.09–0.43, p < 0.001 (**Figure 5B**).

Similar results were also found in the analysis of AFP and MVI, as the OS in the combination group was longer than that in the TACE group irrespective of the stratification of AFP or MVI (**Figures S1** **,** **2**).

### Subsequent Therapy After Progression

As shown in **Table 5**, 23 patients (54.8%) received subsequent treatments in the combination group while 31 patients (75.6%) did in the TACE group. In the TACE group, many patients (n = 18, 43.9%) still chose TACE while only 8 patients (19.5%) received systemic therapy. In the combination group, 23 (54.8%) patients opted for systemic-therapy-based treatment, with 14 of them continuing Lenvatinib combined with PD-1 inhibitor due to the limited treatment options after progression.

**Table 5:** Subsequent treatment after progression.

	Combination Group	TACE Group
	(N = 42) No. (%)	(N = 41) No. (%)
**Accepted Subsequent Treatments**	**23 (54.8)**	**31 (75.6)**
Lenvatinib+PD-1 inhibitor	14 (33.3)	0
Apatinib+PD-1 inhibitor	4 (9.5)	0
Apatinib	3 (7.1)	2 (4.9)
Radiotherapy +PD-1 inhibitor	1 (2.4)	1 (2.4)
Ablation+ PD-1 inhibitor	1 (2.4)	0
Sorafenib	0	5 (12.2)
TACE	0	18 (43.9)
Radiotherapy	0	2 (4.9)
TACE+Radiotherapy	0	3 (7.3)
**Best Supportive Care**	**19 (45.2)**	**10 (24.4)**

The bold values highlighted the factors with significant difference.

Data are presented as n (%).

TACE, transcatheter arterial chemoembolization; PD-1, programmed cell death-1.

### Safety Assessment

All reported AEs were evaluated and found to be mild and tolerable, and no toxicity-related deaths occurred in this study. More Grade 1–2 AEs were found in the combination group, and only hypertension (Grade 3–4 AEs) was more frequent in the combination group than that in the TACE group (17.9% vs. 0,χ^2^ = 8.556, p = 0.003). The details of AEs are summarized in **Table 6**. Immune-related AEs included diabetes with Grade 1 in 2 patients, decreased PLT counts with Grade 3 in 1 patient, and hepatitis with Grade 2 in 1 patient. The decreased PLT count and hepatitis were resolved after treatment with oral corticosteroids at a dose of 1 mg/kg/day.

**Table 6:** Treatment-related adverse events.

Adverse events	Any Grade			Grade 3/4		
	Combination Group (n = 56)	TACE Group (n = 54)	P value	Combination Group (n = 56)	TACE Group (n = 54)	P value
**Treatment-related AEs, n (%)**						
Hypertension	25 (44.6)	0	<0.001	10 (17.9)	0	0.003
Weight loss	20 (35.7)	8 (14.8)	0.012	3 (5.4)	1 (1.9)	0.637
Diarrhea	17 (30.4)	0	<0.001	2 (3.6)	0	0.492
Appetite decreased	16 (28.6)	2 (3.7)	<0.001	2 (3.6)	0	0.492
Fatigue	20 (35.7)	3 (5.6)	<0.001	2 (3.6)	0	0.492
Rash	7 (12.5)	0	0.022	0	0	–
HFSR	12 (21.4)	0	<0.001	0	0	–
Pruritus	6 (10.7)	0	0.040	0	0	–
Hypothyroidism	5 (8.9)	0	0.074	0	0	–
Gum bleeding	3 (5.4)	0	0.255	0	0	–
Constipation	8 (14.3)	2 (3.7)	0.110	0	0	–
Diabete	2 (3.6)	0	0.492	0	0	–
Biloma	0	2 (3.7)	0.459	0	2 (3.7)	0.459
Arthralgia	3 (5.4)	0	0.255	0	0	–
Hoarseness	4 (7.1)	0	0.136	0	0	–
**laboratory-related AEs, n (%)**						
Proteinuria	10 (17.9)	0	0.003	0	0	–
Decreased WBC	20 (35.7)	7 (13.3)	0.006	3 (5.4)	1 (1.9)	0.637
Decreased PLT	23 (41.1)	12 (22.2)	0.034	3 (5.4)	1 (1.9)	0.637
Elevated TB	9 (16.1)	12 (22.2)	0.412	1 (1.8)	2 (3.7)	0.975
Decreased ALB	25 (44.6)	21 (38.9)	0.541	2 (3.6)	2 (3.7)	1.000
Elevated ALT	16 (28.6)	20 (37.0)	0.344	1 (1.8)	3 (5.6)	0.585
Elevated AST	19 (33.9)	24 (44.4)	0.258	1 (1.8)	3 (5.6)	0.585
Elevated GGT	15 (26.8)	20 (37.0)	0.249	0	3 (5.6)	0.229
Elevated Cr	2 (3.6)	0	0.492	0	0	–

p-value was calculated by a two-sided Chi-square test.

HFSR, hand-foot skin reaction; WBC, white blood cell; PLT, platelet; TB, total bilirubin; ALB, albumin; ALT, alanine aminotransferase; AST, aspartate aminotransferase; GGT, gamma-glutamyl transferase; Cr, creatinine.

## Discussion

In this study, we found that TACE combined with Lenvatinib plus a PD-1 inhibitor achieved more favorable results than TACE alone in patients with uHCC. Although there was a high percentage of patients with advanced stage disease (BCLC C 69.6%), MVI (33.9%), and extrahepatic metastasis (EHS) (51.8%) among those treated with the combination therapy, they had a higher rate of ORR and better survival benefits than those treated with the control therapy (ORR 67.9 vs. 29.6%; mPFS 11.9 vs. 6.9 months; mOS 23.9 vs. 15.3 months). Some of these patients even were found to have an early and quick response after only 1–2 cycles of combination therapy, leading to the normalization of tumor markers, shrinkage and fusion necrosis of tumors, disappearance of tumor daughter nodules, and hypertrophy of the future liver remnant, which may increase the possibility of a conversion hepatectomy in patients initially diagnosed with uHCC. Therefore, the triple combination treatment of TACE-Lenvatinib-PD-1 inhibitors might be an effective and promising treatment strategy for uHCC patients.

Studies on TKIs in combination with ICIs in patients with uHCC proved the potential synergetic effect. TKI and ICI monotherapies were shown to have limited efficacy in clinical studies, with a limited ORR of 9.2% in Sorafenib (3), 17% in PD-1 inhibitors (23), and 24.1% in Lenvatinib (3); however, the combination therapy of TKIs and ICIs were found to improve the survival significantly. An ORR of 33.2% was reported in the IMbrave 150 study with the combination of atezolizumab plus bevacizumab (18) and a higher ORR of 46% was reported in the Keynote 524 study with pembrolizumab plus Lenvatinib (19). These indicated that the combination treatment may have synergistic antitumor effects. The predominant effects not only mainly depend on the role of the PD-1/PD-L1 interaction but are also significantly affected by the multiple roles of anti-angiogenesis drugs. The Anti-VEGF functions were found to be involved in several steps of T-cell activation, including the restoration of antigen presentation, the priming and activation of T-cell responses, and the modulation of the tumor immune microenvironment (24, 25). Furthermore, Lenvatinib was also found to have other pathways in the modulation of antitumor immunity, including the reduction of tumor PD-L1 expression levels and Treg differentiation by blocking FGFR4 (26) and reducing the Treg proportion through TGF-β pathway inhibition (27).

In this triple combination group, patients achieved a 67.9% ORR according to the mRECIST criteria, which significantly higher than that of dual combination, with an ORR of 33.2-46% (18, 19, 28) in PD-1/PD-L1 inhibitors combined with antiangiogenics, and 27.8-53.1% (29, 30) in TACE combined with Lenvatinib. It has been found that the application of TACE alongside TKI and ICI resulted in more synergistic antitumor effects. In addition to the conventional role of tumor necrosis leading by TACE (31), TACE was found to promote T-cell activation *via* abscopal effects. The tumor necrosis caused by TACE increased the release of tumor-associated antigens (32), which has been proven to recruit DCs (33), increase AFP-specific CD4+T-cell response (13), synergize with ICIs to increase cytotoxic T lymphocytes, and decrease tumor-infiltrating Treg cells (34). TKI (Lenvatinib) was also found to effectively inhibit the angiogenic growth factors triggered by the extensive ischemic necrosis in preclinical (15) or clinical studies (16, 17), which was also found to be an important factor associated with T-cell activation. These synergistic mechanisms might contribute to the favorable clinical outcomes. Recent studies have also demonstrated the encouraging clinical data of TKI and PD-1 inhibitor in combination with transarterial therapy with an ORR of 75.7-84.2% and a DCR of 86.5-94.7% (35, 36). The results of our study are comparable and might offer more evidence of this triple combination treatment approach for uHCC patients.

Upon multivariate analysis, NLR was found to be an independent prognostic factors associated with both OS and PFS. Several studied found NLR in peripheral blood significantly affected the survival in patients with uHCC treated with systemic therapy (37, 38). NLR reflects a potential balance between neutrophil-associated protumor inflammation and lymphocyte-dependent antitumor immune response. Based on the cutoff value of generally 3.0–4.5 (39), patients were divided into two groups with different responses to ICI (40). The exploration results in our study also demonstrated these findings. Patients with low NLR had a significant response to combination therapy and longer survival than those in the TACE group (mOS 28.9 vs. 15.2 months, p < 0.001). On the other hand, in those with high NLR, survival did not differ significantly between the combination group and the TACE group (mOS 17.3 vs. 15.5 months, p = 0.2013). Thus, NLR might be a noninvasive and predictive indicator to identify patients who may benefit from combination therapy.

A tolerable safety profile was observed with combination therapy in this study. There were no unexceptional toxicities in triple treatment group. The frequent AEs with any-grade AEs included hypertension, decreased ALB, and decreased PLT, in combination therapy. Moreover, the most common Grade 3 or Grade 4 AE in combination group was hypertension, which could be manageable by dose reduction or antihypertension drugs without treatment discontinuation. The tolerable safety profiles guaranteed the long-term medication to achieve survival benefits.

However, this study had several limitations. Firstly, it was a prospective, observational, cohort study. The patients selected the treatment strategy after being fully informed of the efficacy, potential AEs, and cost of each drug. The various selection biases and potential differences in baseline characteristics would affect the treatment results. Secondly, the sample size of our study was small. The enrolled patients with uHCC were heterogeneous, including patients at BCLC B and C stages, even though combination therapy could bring potential benefits for either B-stage or C-stage HCC patients; thus, the results of this study require further confirmation. Thirdly, the control group of our study enrolled patients who took only TACE. It was the recommended treatment for BCLC C-stage HCC patients in Chinese guidelines and could offer some survival benefits from the control of local lesions. However, it might have some bias on the results. Thus, the results should be interpreted cautiously. More large-scale, randomized, controlled clinical trials are needed to confirm the efficacy of triple combination therapy.

In conclusion, our study demonstrated that compared with TACE alone, combination therapy with TACE and Lenvatinib plus a PD-1 inhibitor might be associated with better survival benefits with manageable toxicity profiles and the magnitude of benefit was significantly more intense in patients with low NLR who received combination therapy.

## Data Availability Statement

The raw data supporting the conclusions of this article will be made available by the authors, without undue reservation.

## Ethics Statement

The studies involving human participants were reviewed and approved by The ethical committee of the Third Affiliated Hospital of the Second Military Medical University. The patients/participants provided their written informed consent to participate in this study. Written informed consent was obtained from the individual(s) for the publication of any potentially identifiable images or data included in this article.

## Author Contributions

SQ, XZ, LW, and DW were involved in the conception and design of the study. DW, LH, JZ, and RW were involved in the provision of study materials and the recruitment of patients. SQ, XZ, YW, YM, HP, and QF were involved in data acquisition and assembly. SQ, LH, JZ, RW, LW, and DW were involved in data analysis and interpretation. SQ, XZ, LW, and DW were involved in manuscript writing. All authors contributed to the article and approved the submitted version.

## Funding

This work was supported by a grant from the State Key Project on Infectious Diseases of China (2018ZX10723204), the National Natural Science Foundation of China (Grant NO. 81630070), the Program of Science and Technology Commission of the Shanghai Municipality (18411969200).

## Conflict of Interest

The authors declare that the research was conducted in the absence of any commercial or financial relationships that could be construed as a potential conflict of interest.

The handling editor JY declared a shared parent affiliation with the authors SQ, XZ, YM, HP, QF, LH, JZ, RW, LW, and DW at the time of review.

## Publisher’s Note

All claims expressed in this article are solely those of the authors and do not necessarily represent those of their affiliated organizations, or those of the publisher, the editors and the reviewers. Any product that may be evaluated in this article, or claim that may be made by its manufacturer, is not guaranteed or endorsed by the publisher.
